# Supplementary material for: Xanthomonas effector XopR hijacks host actin cytoskeleton via complex coacervation
Source: Nat Commun. 2021 Jul 1;12:4064. doi: 10.1038/s41467-021-24375-3 (PMC8249405; doi:10.1038/s41467-021-24375-3)
Supplement: Supplementary file 1 — Supplementary Information [file 41467_2021_24375_MOESM1_ESM.pdf]

Supplementary Information

Xanthomonas Effector XopR Hijacks Host Actin Cytoskeleton Via Complex Coacervation

He Sun[1], Xinlu Zhu[1], Chuanxi Li[2], Zhiming Ma[1], Xiao Han[1], Yuanyuan Luo[1], Liang Yang[1,3], Jing Yu[2], Yansong Miao[1 *]

[1]School of Biological Sciences, Nanyang Technological University, Singapore 637551, Singapore
[2]School of Materials Science and Engineering, Nanyang Technological University, Singapore 639798, Singapore
[3]Singapore Centre for Environmental Life Sciences Engineering, Nanyang Technological University, Singapore 637551, Singapore
*Correspondence: yansongm@ntu.edu.sg

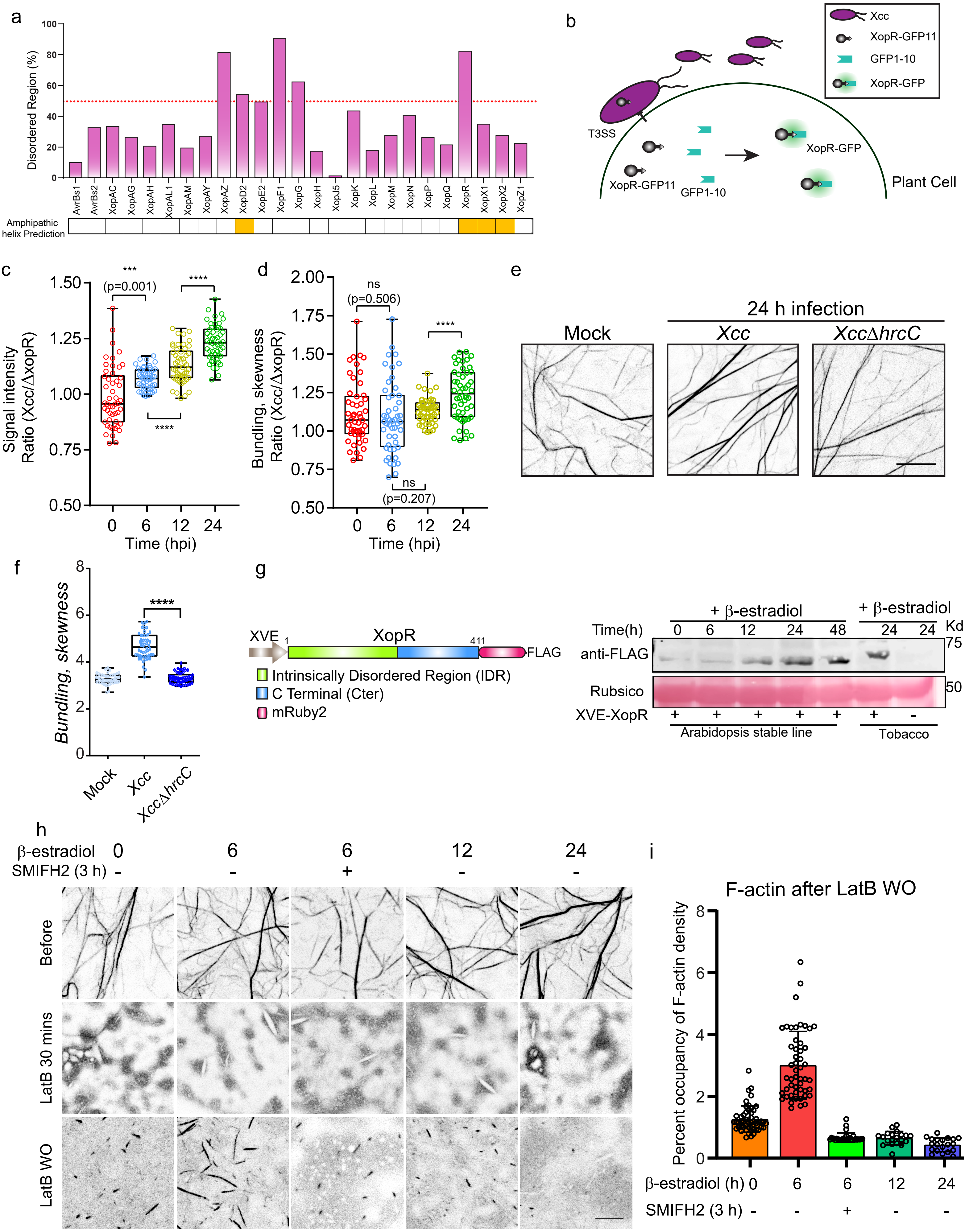
a
Disordered Region (%)
Amphipathic helix Prediction
AvrBs1
AvrBs2
XopAC
XopAG
XopAH
XopAL1
XopAM
XopAY
XopAZ
XopD2
XopE2
XopF1
XopG
XopH
XopJ5
XopK
XopL
XopM
XopN
XopP
XopQ
XopR
XopX1
XopX2
XopZ1
b
Xcc
XopR-GFP11
GFP1-10
XopR-GFP
T3SS
Plant Cell
c
Signal intensity Ratio (Xcc/ΔxopR)
(p=0.001)
Time (hpi)
d
Bundling, skewness Ratio (Xcc/ΔxopR)
ns
(p=0.506)
(p=0.207)
Time (hpi)
e
24 h infection
Mock
Xcc
XccΔhrcC
f
Bundling, skewness
Mock
Xcc
XccΔhrcC
g
XVE
XopR
FLAG
Intrinsically Disordered Region (IDR)
C Terminal (Cter)
mRuby2
+ β-estradiol
Time(h)
anti-FLAG
Rubisco
XVE-XopR
Arabidopsis stable line
Tobacco
Kd
h
β-estradiol
SMIFH2 (3 h)
Before
LatB 30 mins
LatB WO
i
F-actin after LatB WO
Percent occupancy of F-actin density
β-estradiol (h)
SMIFH2 (3 h)

Supplementary Fig. 1 XopR Remodels the host actin cytoskeleton in a dose-dependent manner.

a, Intrinsically disordered region prediction and amphipathic helix of *Xanthomonas campestris* pv. *Campestris (Xcc)* type III effectors by the IUPRED2 algorithm and NPS@:AMPHIPASEEK. **b**, Cartoon illustration of the split GFP system for monitoring the real-time injection of XopR. XopR was tagged with GFP11 in the Xcc genome, 35S:: GFP1-10 Arabidopsis transgenic line was used to perform pathogen infection. **c,d**, Quantification of ratio changes in Lifeact-Venus intensity and actin-bundling, respectively, between *Xcc* and *XccΔxopR* infected seedlings (n = 50 images from 10 individual seedlings, box plots represent mean with SD and whiskers represent min to max). **e,f**, Representative Lifeact-Venus images and bundling measurements from Arabidopsis seedlings infected by *Xcc* and *XccΔhrcC* inoculation for 24 h (n = 50 images from 10 individual seedlings, box plots represent mean with SD and whiskers represent min to max). **g**, (Left) Schematic illustration of XVE-XopR-Ruby2 constructs for transgenic Arabidopsis. (Right) Western blot detection of XopR in total protein extracts from leaf discs from 1-month-old pXVE::XopR-mRuby2-FLAG plants when treated with 10 μM β-estradiol for the indicated time, and the *N. benthamiana* epidermal cells transiently with or without expressing XopR-FL-mRuby2. Ponceau red-stained rubisco large subunit was used as the loading control. **h,i**, Representative images of Lifeact-Venus and its percent occupancy measurement in the LatB washout assay, with or without formin inhibitor SMIFH2. Seven-day-old Arabidopsis seedlings expressing pXVE::XopR-mRuby2-FLAG were applied with 10 μM β-estradiol for the indicated time before subjection to 5 μM LatB treatment for another 30 min. 50 μM SMIFH2 was added during the first 6 h of β-estradiol induction for a 3 h incubation prior to LatB treatment and washout. Images were taken after 30 minutes following LatB washout. (From left to right, n = 50, 50, 30, 20, 20 images, each from five individual seedlings in two independent experiments). Error bar, SD. Two-tailed Student's t-test was performed assuming equal variance. ns = no significant difference, *p<0.05, **p<0.01, ***p<0.001, ****p<0.0001. Scale bar = 10 μm for **e**, 5 μm for **h.**

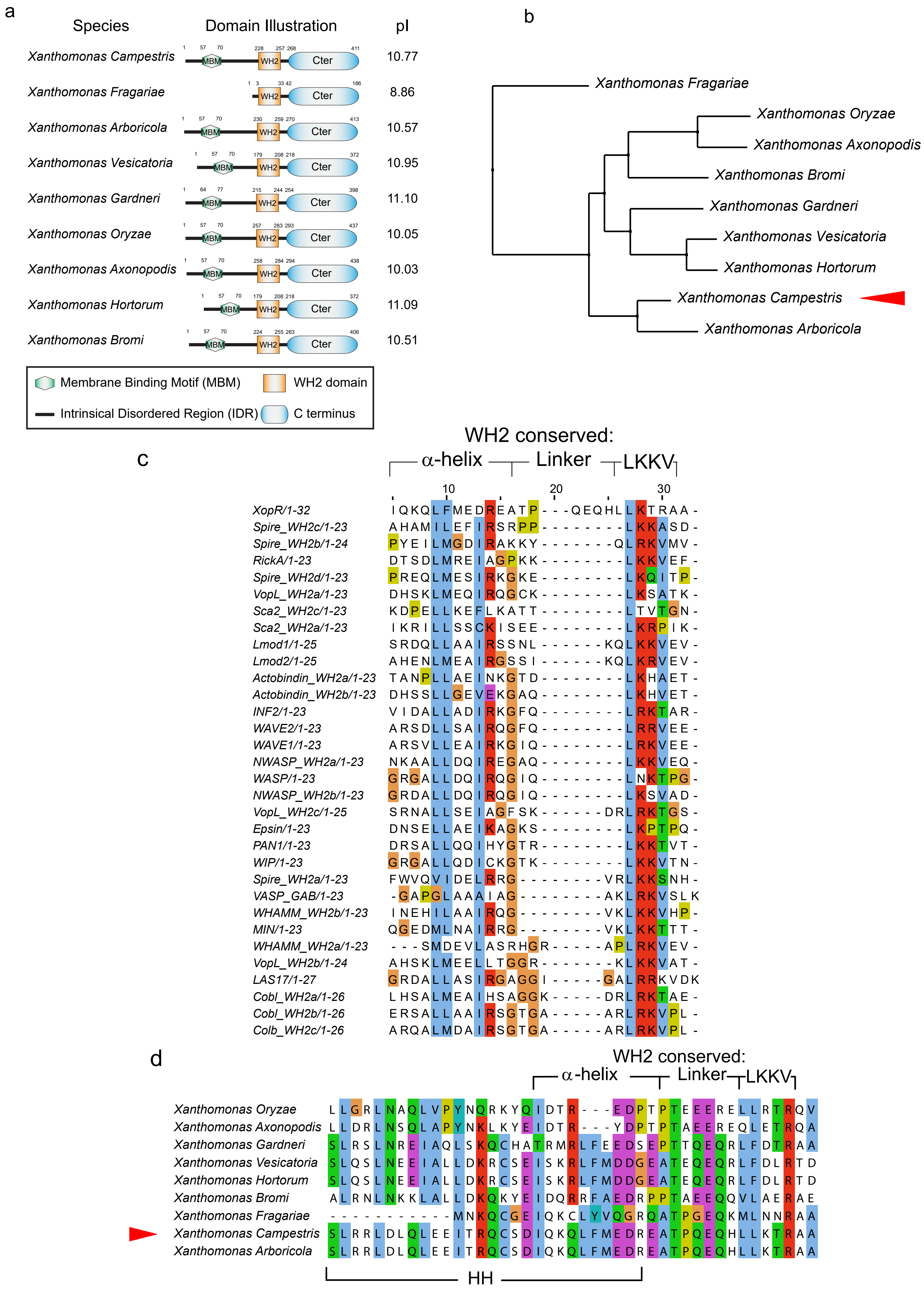

a
Species
Domain Illustration
pI
Xanthomonas Campestris
1 57 70 228 257 268 411
MBM
WH2
Cter
10.77
Xanthomonas Fragariae
1 3 33 42 186
WH2
Cter
8.86
Xanthomonas Arboricola
1 57 70 230 259 270 413
MBM
WH2
Cter
10.57
Xanthomonas Vesicatoria
1 57 70 179 208 218 372
MBM
WH2
Cter
10.95
Xanthomonas Gardneri
1 64 77 215 244 254 398
MBM
WH2
Cter
11.10
Xanthomonas Oryzae
1 57 70 257 283 293 437
MBM
WH2
Cter
10.05
Xanthomonas Axonopodis
1 57 70 258 284 294 438
MBM
WH2
Cter
10.03
Xanthomonas Hortorum
1 57 70 179 208 218 372
MBM
WH2
Cter
11.09
Xanthomonas Bromi
1 57 70 224 255 263 406
MBM
WH2
Cter
10.51
Membrane Binding Motif (MBM)
WH2 domain
Intrinsical Disordered Region (IDR)
C terminus
b
Xanthomonas Fragariae
Xanthomonas Oryzae
Xanthomonas Axonopodis
Xanthomonas Bromi
Xanthomonas Gardneri
Xanthomonas Vesicatoria
Xanthomonas Hortorum
Xanthomonas Campestris
Xanthomonas Arboricola
c
WH2 conserved:
α-helix
Linker
LKKV
10 20 30
XopR/1-32 IQKQLFMEDREATP---QEQHLLKTRAA-
Spire_WH2c/1-23 AHAMILEFIRSRPP--------LKKASD-
Spire_WH2b/1-24 PYEILMGDIRAKKY-------QLRKVMV-
RickA/1-23 DTSDLMREIAGPKK--------LKKVEF-
Spire_WH2d/1-23 PREQLMESIRKGKE--------LKQITP-
VopL_WH2a/1-23 DHSKLMEQIRQGCK--------LKSATK-
Sca2_WH2c/1-23 KDPELLKEFLKATT--------LTVTGN-
Sca2_WH2a/1-23 IKRILLSSCKISEE--------LKRPIK-
Lmod1/1-25 SRDQLLAAIRSSNL------KQLKKVEV-
Lmod2/1-25 AHENLMEAIRGSSI------KQLKRVEV-
Actobindin_WH2a/1-23 TANPLLAEINKGTD--------LKHAET-
Actobindin_WH2b/1-23 DHSSLLGEVEKGAQ--------LKHVET-
INF2/1-23 VIDALLADIRKGFQ--------LRKTAR-
WAVE2/1-23 ARSDLLSAIRQGFQ--------LRRVEE-
WAVE1/1-23 ARSVLLEAIRKGIQ--------LRKVEE-
NWASP_WH2a/1-23 NKAALLDQIREGAQ--------LKKVEQ-
WASP/1-23 GRGALLDQIRQGIQ--------LNKTPG-
NWASP_WH2b/1-23 GRDALLDQIRQGIQ--------LKSVAD-
VopL_WH2c/1-25 SRNALLSEIAGFSK------DRLRKTGS-
Epsin/1-23 DNSELLAEIKAGKS--------LKPTPQ-
PAN1/1-23 DRSALLQQIHYGTR--------LKKTVT-
WIP/1-23 GRGALLQDICKGTK--------LKKVTN-
Spire_WH2a/1-23 FWVQVIDELRRG--------VRLKKSNH-
VASP_GAB/1-23 -GAPGLAAAIAG--------AKLRKVSLK
WHAMM_WH2b/1-23 INEHILAAIRQG--------VKLKKVHP-
MIN/1-23 QGEDMLNAIRRG--------VKLKKTTT-
WHAMM_WH2a/1-23 ---SMDEVLASRHGR-----APLRKVEV-
VopL_WH2b/1-24 AHSKLMEELLTGGR-------KLKKVAT-
LAS17/1-27 GRDALLASIRGAGGI-----GALRRKVDK
Cobl_WH2a/1-26 LHSALMEAIHSAGGK-----DRLRKTAE-
Cobl_WH2b/1-26 ERSALLAAIRSGTGA-----ARLRKVPL-
Colb_WH2c/1-26 ARQALMDAIRSGTGA-----ARLRKVPL-
d
WH2 conserved:
α-helix
Linker
LKKV
Xanthomonas Oryzae LLGRLNAQLVPYNQRKYQIDTR---EDPTPTEEERELLRTRQV
Xanthomonas Axonopodis LLDRLNSQLAPYNKLKYEIDTR---YDPTPTAEEREQLETRQA
Xanthomonas Gardneri SLRSLNREIAQLSKQCHATRMRLFEEDSEPTTQEQRLFDTRAA
Xanthomonas Vesicatoria SLQSLNEEIALLDKRCSEISKRLFMDDGEATEQEQRLFDLRTD
Xanthomonas Hortorum SLQSLNEEIALLDKRCSEISKRLFMDDGEATEQEQRLFDLRTD
Xanthomonas Bromi ALRNLNKKLALLDKQKYEIDQRRFAEDRPPTAEEQQVLAERAE
Xanthomonas Fragariae -----------MNKQCGEIQKCLYVQGRQATPGEQKMLNNRAA
Xanthomonas Campestris SLRRLDLQLEEITRQCSDIQKQLFMEDREATPQEQHLLKTRAA
Xanthomonas Arboricola SLRRLDLQLEEITRQCSDIQKQLFMEDREATPQEQHLLKTRAA
HH

Supplementary Fig. 2 Sequence alignment and evolutionary analysis of XopR.

a, Schematic illustration of the XopR domains among *Xanthomonas* homologs. The membrane binding motif was predicted online via NPS@:AMPHIPASE EK. The WH2 domain and isoelectric point (pI) are indicated. **b**, Phylogenetic tree of XopR homologs among *Xanthomonas* species. **c,d**, Alignments of the WH2 domains and WH2-related sequences via the Clustal Omega, among known WH2 domain-containing proteins **c** and *Xanthomonas* species **d**.

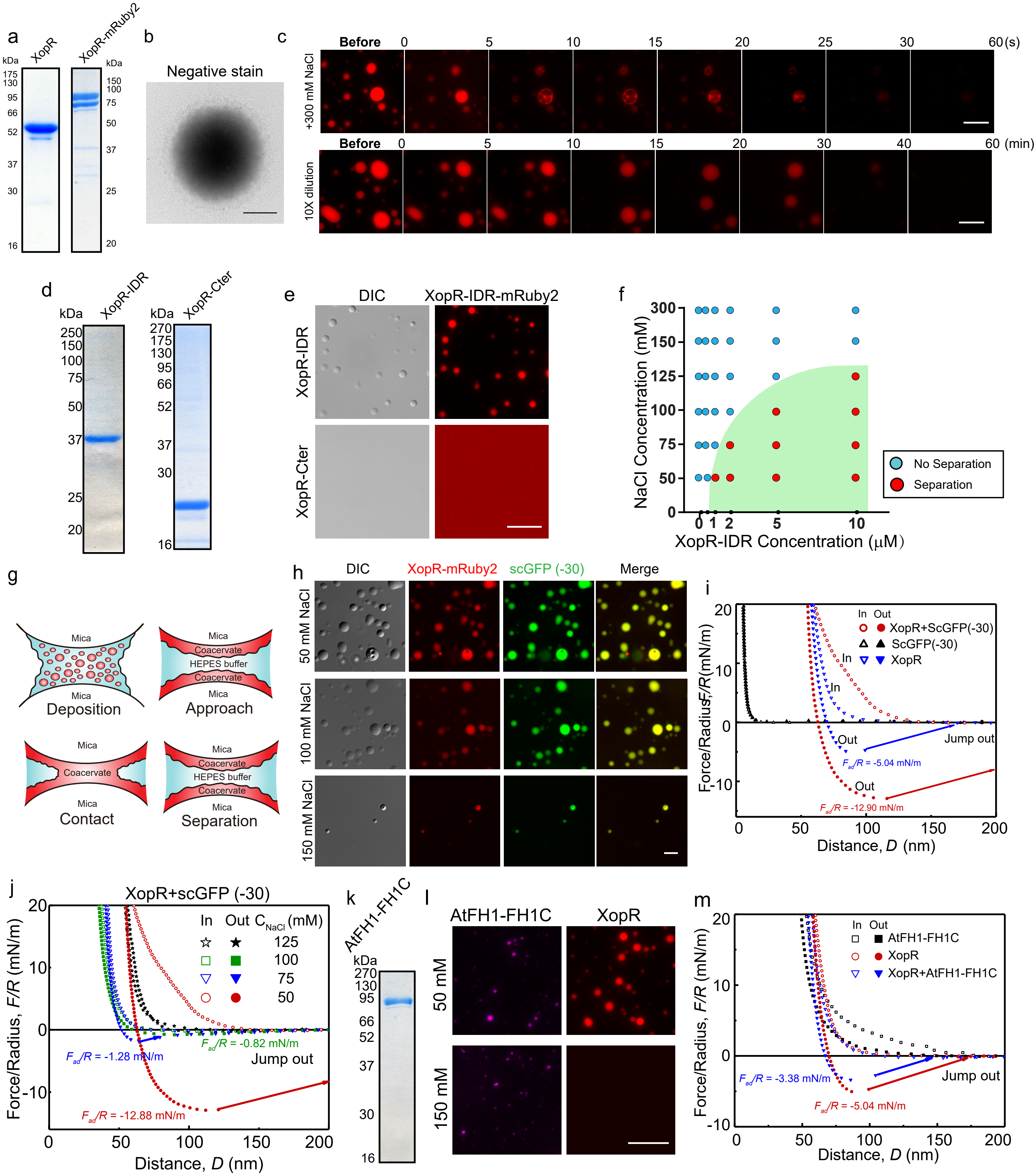
a
XopR
XopR-mRuby2
kDa
b
Negative stain
c
+300 mM NaCl
Before
0 5 10 15 20 25 30 60(s)
10X dilution
0 5 10 15 20 30 40 60 (min)
d
XopR-IDR
XopR-Cter
kDa
e
DIC
XopR-IDR-mRuby2
XopR-IDR
XopR-Cter
f
NaCl Concentration (mM)
XopR-IDR Concentration (μM)
No Separation
Separation
g
Mica
Coacervate
HEPES buffer
Deposition
Approach
Contact
Separation
h
DIC
XopR-mRuby2
scGFP (-30)
Merge
50 mM NaCl
100 mM NaCl
150 mM NaCl
i
In Out
XopR+ScGFP(-30)
ScGFP(-30)
XopR
Force/Radius, F/R (mN/m)
Distance, D (nm)
Jump out
F_ad/R = -5.04 mN/m
F_ad/R = -12.90 mN/m
j
XopR+scGFP (-30)
In Out C_NaCl (mM)
125
100
75
50
F_ad/R = -1.28 mN/m
F_ad/R = -0.82 mN/m
F_ad/R = -12.88 mN/m
Jump out
Force/Radius, F/R (mN/m)
Distance, D (nm)
k
AtFH1-FH1C
kDa
l
AtFH1-FH1C
XopR
50 mM
150 mM
m
In Out
AtFH1-FH1C
XopR
XopR+AtFH1-FH1C
F_ad/R = -3.38 mN/m
F_ad/R = -5.04 mN/m
Jump out
Force/Radius, F/R (mN/m)
Distance, D (nm)

Supplementary Fig. 3 Biochemical and biophysical characterization of XopR coacervates.

a, Coomassie-blue staining of recombinant XopR and XopR-mRuby2 on SDS-PAGE. **b**, Negative stained TEM of XopR droplet from 10 μM XopR in a low salt buffer (20mM HEPES, 50 mM NaCl, pH =7.4). **c**, Reversible transitions of XopR LLPS by increasing the NaCl concentration to 200 mM (upper panel) or diluting the XopR concentration to 1/10 (bottom panel). **d**, Coomassie-blue staining of recombinant XopR-IDR and XopR-Cter by SDS-PAGE. **e**, Droplet formation of XopR-IDR (top) or XopR-Cter (bottom) in low salt buffer (20 mM HEPES, 150 mM NaCl, pH=7.4). For this experiment, 10 μM of XopR-IDR (10% XopR-IDR-mRuby2) or XopR-Cter (10% XopR-Cter-mRuby2) was prepared in the low salt buffer] for 10 mins before imaging. **f**, Phase diagram of XopR-IDR (XopR-IDR concentration versus NaCl concentration). Phase diagrams were generated from at least 10 images for each condition. **g**, Schematic of the adhesion experiment: (Deposition) coacervate settling onto a mica surface; (Approach) formation of a uniform coacervate film on mica surface; (Contact) approaching of two surfaces and formation of a uniform coacervate bridge between two mica surfaces; and (Separation) after separation and breakage of the coacervate neck. **h**, XopR (5 μM, 10% XopR-mRuby2) was incubated with 5 μM of scGFP(-30) in the indicated concentration of NaCl solution for 10 mins before imaging. **i,j**, Force-distance profiles are relative to the separation distance between two surfaces with XopR, scGFP(-30), or XopR-scGFP(-30) in 50 mM NaCl buffer, as well as XopR-scGFP(-30) at different salt concentrations. **k**, Coomassie-blue staining of recombinant AtFH1-FH1C by SDS-PAGE. **l**, XopR (5 μM, 10% XopR-mRuby2) and AtFH1-FH1C (5 μM, 10% Alexa647-AtFH1-FH1C) form droplets in the low salt buffer (20 mM HEPES, 50 mM NaCl, pH=7.4) and physiological buffer (20 mM HEPES, 150 mM NaCl, pH=7.4), respectively. **m**, Force–distance profiles relative to the separation distance between two surfaces with XopR or AtFH1-FH1C in 20 mM HEPES, 50mM NaCl buffer. Scale bar = 500 nm for **m** and 10 μm for (**c, e, h**, and **l**).

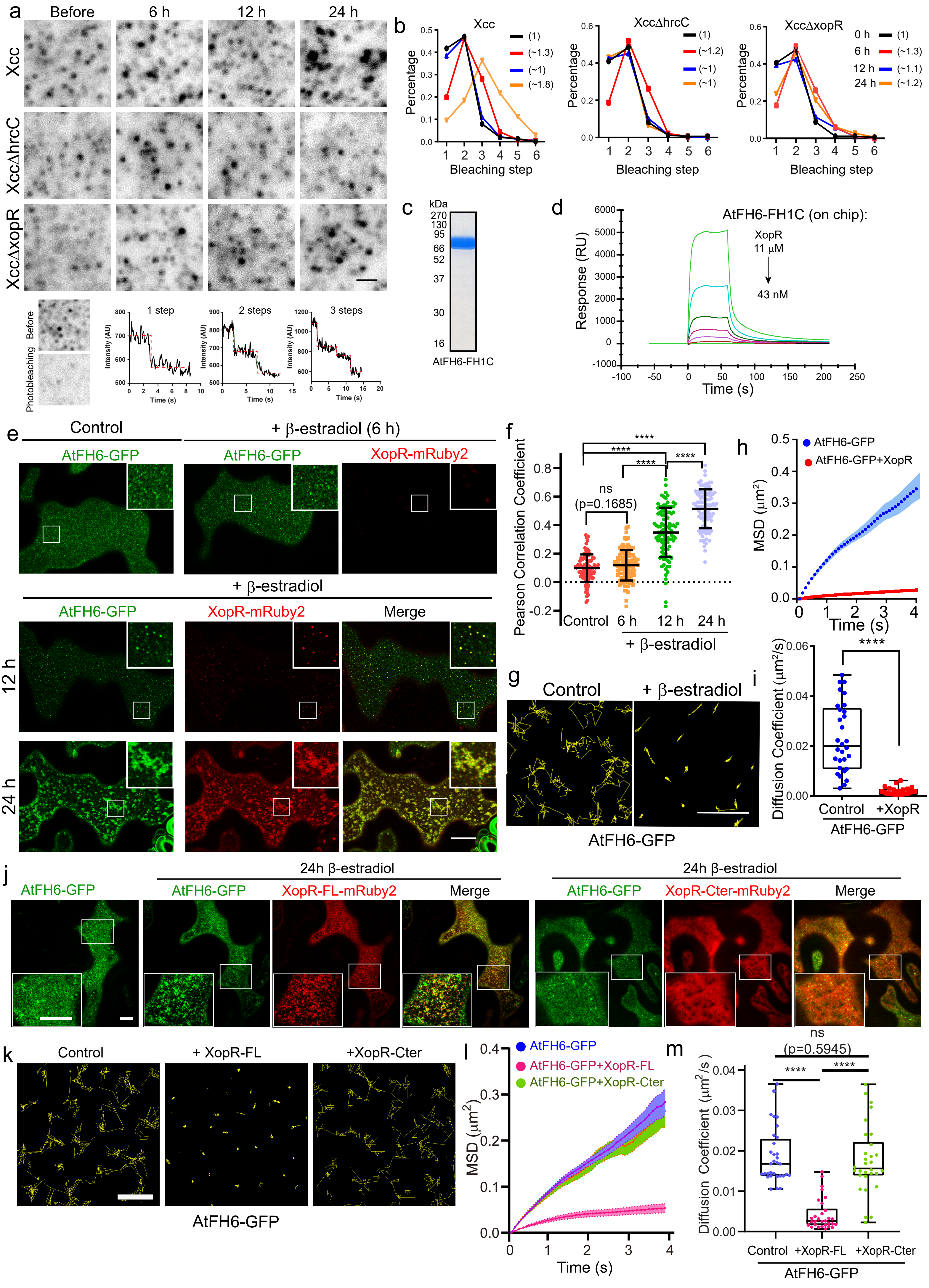
a
Before
6 h
12 h
24 h
Xcc
XccΔhrcC
XccΔxopR
Before
Photobleaching
1 step
2 steps
3 steps
Intensity (AU)
Time (s)
b
Xcc
XccΔhrcC
XccΔxopR
Percentage
Bleaching step
0 h (1)
6 h (~1.3)
12 h (~1)
24 h (~1.8)
(~1.2)
(~1)
(~1)
(~1.3)
(~1.1)
(~1.2)
c
kDa
270
130
95
66
52
37
30
16
AtFH6-FH1C
d
AtFH6-FH1C (on chip):
XopR
11 μM
43 nM
Response (RU)
Time (s)
e
Control
+ β-estradiol (6 h)
AtFH6-GFP
XopR-mRuby2
+ β-estradiol
Merge
12 h
24 h
f
Pearson Correlation Coefficient
ns
(p=0.1685)
****
Control
6 h
12 h
24 h
+ β-estradiol
g
Control
+ β-estradiol
AtFH6-GFP
h
AtFH6-GFP
AtFH6-GFP+XopR
MSD (μm²)
Time (s)
i
Diffusion Coefficient (μm²/s)
****
Control
+XopR
AtFH6-GFP
j
AtFH6-GFP
24h β-estradiol
XopR-FL-mRuby2
XopR-Cter-mRuby2
Merge
k
Control
+ XopR-FL
+XopR-Cter
AtFH6-GFP
l
AtFH6-GFP
AtFH6-GFP+XopR-FL
AtFH6-GFP+XopR-Cter
MSD (μm²)
Time (s)
m
ns
(p=0.5945)
****
Diffusion Coefficient (μm²/s)
Control
+XopR-FL
+XopR-Cter
AtFH6-GFP

Supplementary Fig. 4 XopR-mediated formin clustering on the plasma membrane in vivo.

a, Representative VA-TIRF images of the formin single particle bleaching experiment using AtFH6-GFP line in 7-days-old *Arabidopsis* seedlings were dip-inoculated with *Xcc*/ *XccΔ hrcC*/ *XccΔ xopR* (upper panel). Images were taken at 6 hpi, 12 hpi and 24 hpi. Representative single particle images before and after photobleaching, and signal intensity traces during photobleaching were shown (bottom panel). **b**, Distribution analysis of photobleaching step counting for AtFH6-GFP puncta in *Xcc*-infected seedlings (n=151 for 0 hpi, n=206 for 6 hpi, n=182 for 12 hpi, n=167 for 24 hpi), *XccΔhrcC* (n=159 for 0 hpi, n=187 for 6 hpi, n=184 for 12 hpi, n=165 for 24 hpi), and *XccΔxopR* (n=170 for 0 hpi, n=186 for 6 hpi, n=146 for 12 hpi, n=164 for 24 hpi). **c**, Coomassie-blue staining of recombinant AtFH6-FH1C by SDS-PAGE. **d**, Representative SPR sensorgram of XopR binding to AtFH6-FH1C. A serial concentration of AtFH6-FH1C ranging from 43 nM to 11 μM was injected onto the XopR-immobilized chip. **e**, Representative confocal images of AtFH6-GFP at the cell surface in the epidermis of seven-day-old Arabidopsis cotyledons, which was treated with 10 μM β-estradiol to trigger XopR induction under the XVE promoter for 6, 12 or 24 hours before imaging. **f**, Pearson correlation coefficient calculation between XopR-mRuby2 and AtFH6-GFP puncta of **e** (n=100 of 10x10 μm^2 ROIs, Error bar, SD) **g**, Representative trajectory map of mobile AtFH6-GFP foci on the PM with or without XopR 24h induction by 10 μM β-estradiol. **h,i**, MSD and diffusion coefficient analysis of AtFH6-GFP in **g** (n= 25 movies from 5 individual seedlings, data are presented as mean values with error bands which represent SD, box plots represent mean with SD and whiskers represent min to max). **j.** Representative confocal images of *N. benthamiana* epidermal cells transiently expressing AtFH6-GFP with or without XopR-FL-mRuby2 and XopR-Cter-mRuby2. Images were taken after 24h of 20 μM β-estradiol induction. **k**. Representative trajectory maps of AtFH6-GFP on the PM using VA-TIRF imaging for the experiments of (j). **l.m**. MSD and diffusion coefficiency analysis of AtFH6-GFP in **k** (n=30 movies, data are presented as mean values with error bands which represent SEM, box plots represent mean with SD and whiskers represent min to max). Two-tailed Student's t-test was performed. ns = no significant difference, *p<0.05, **p<0.01, ***p<0.001, ****p<0.0001. Scale bar = 1 μm for **a,k**, 20 μm for **e**, 5 μm for **g**, 10 μm for **j**.

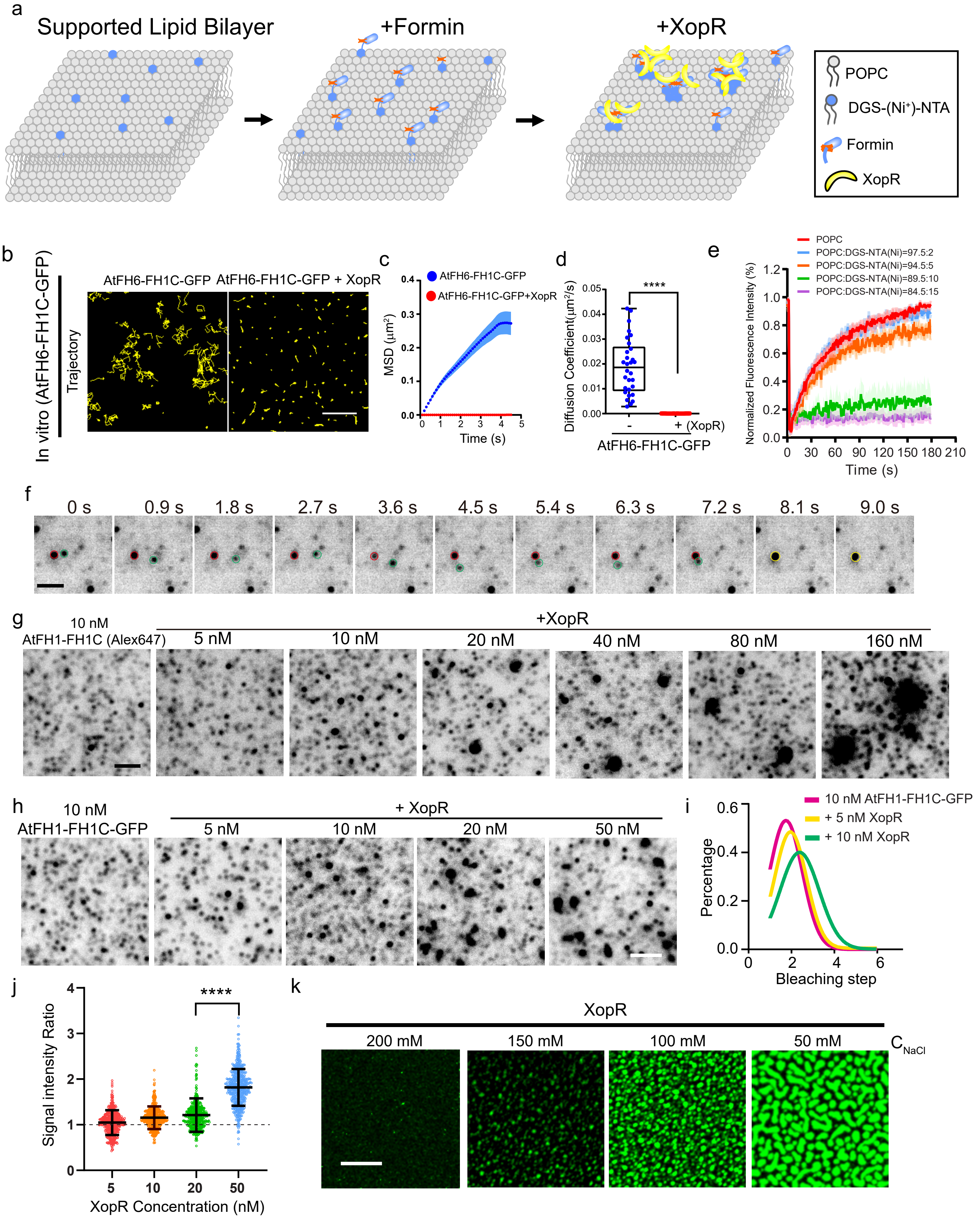

a
Supported Lipid Bilayer
+Formin
+XopR
POPC
DGS-(Ni+)-NTA
Formin
XopR
b
In vitro (AtFH6-FH1C-GFP)
Trajectory
AtFH6-FH1C-GFP
AtFH6-FH1C-GFP + XopR
c
AtFH6-FH1C-GFP
AtFH6-FH1C-GFP+XopR
MSD (μm²)
Time (s)
d
Diffusion Coefficient(μm²/s)
****
- + (XopR)
AtFH6-FH1C-GFP
e
POPC
POPC:DGS-NTA(Ni)=97.5:2
POPC:DGS-NTA(Ni)=94.5:5
POPC:DGS-NTA(Ni)=89.5:10
POPC:DGS-NTA(Ni)=84.5:15
Normalized Fluorescence Intensity (%)
Time (s)
f
0 s
0.9 s
1.8 s
2.7 s
3.6 s
4.5 s
5.4 s
6.3 s
7.2 s
8.1 s
9.0 s
g
10 nM
AtFH1-FH1C (Alex647)
+XopR
5 nM
10 nM
20 nM
40 nM
80 nM
160 nM
h
10 nM
AtFH1-FH1C-GFP
+ XopR
5 nM
10 nM
20 nM
50 nM
i
10 nM AtFH1-FH1C-GFP
+ 5 nM XopR
+ 10 nM XopR
Percentage
Bleaching step
j
****
Signal intensity Ratio
XopR Concentration (nM)
k
XopR
200 mM
150 mM
100 mM
50 mM
C_NaCl

Supplementary Fig. 5 XopR-mediated formin clustering on the SLB in vitro.

a, Cartoon illustration of the SLB-based reconstitution assay. **b**, Representative moving trajectory of AtFH6-FH1C-GFP foci on a reconstituted SLB. AtFH6-FH1C-GFP was supplied onto SLB (98% POPC+ 2% DGS-NTA-Ni^{+}) for 5 min before imaging. **c,d**, MSD and diffusion coefficient analysis of AtFH6-FH1C-GFP in **b** (n= 25 movies, data are presented as mean values with error bands which represent SD, box plots represent mean with SD and whiskers represent min to max). **e**, FRAP of SLBs that were generated at the indicated compositions, in which 0.5% DPPE-Rhodamine were used FRAP (n=6 for each FRAP, error bar represents SEM). **f**, Time-lapse images showing XopR mediated fusion of AtFH1-FH1C. 30 nM AtFH1-FH1C (50% Alexa 647 labeled AtFH1-FH1C) was incubated on dynamic SLB (98% POPC+ 2% DGS-NTA-Ni^{+}) for 1 min to final density as ~40 molecules/100 μm^2. The free AtFH1-FH1C was washed away before adding 100 nM XopR into the solution, which was immediately followed by TIRFM imaging. Representative images show two AtFH1-FH1C particles (marked as red and green) fused to a single particle (marked as yellow). **g**, Representative TIRF images of AtFH1-FH1C (10 nM, 50% Alex-647 labeled) with different concentrations of XopR on immobilized SLB (80% POPC+ 20% DGS-NTA-Ni^{+}). **h**, Representative TIRF images of 10 nM AtFH1-FH1C-GFP with different concentrations of XopR on the stabilized-SLB. Scale bar = 2 μm. **i**, Distribution of bleaching step for AtFH1-FH1C-GFP puncta with different concentrations of XopR (n=100 puncta for each condition). **j**, AtFH1-FH1C-GFP puncta signal intensity ratio after adding indicated concentrations of XopR comparing to AtFH1-FH1C-GFP alone. n=500; Error bar, SD. **k**, Representative dual-color confocal images 5 μM XopR (10% Alexa488-XopR) on SLB with the indicated concentration of NaCl. Scale bar = 2 μm for **b,f**. Scale bar = 10 μm for **k**. Two-tailed Student's t-test was performed assuming equal variance. Ns = no significant difference, *p<0.05, **p<0.01, ***p<0.001, ****p<0.0001

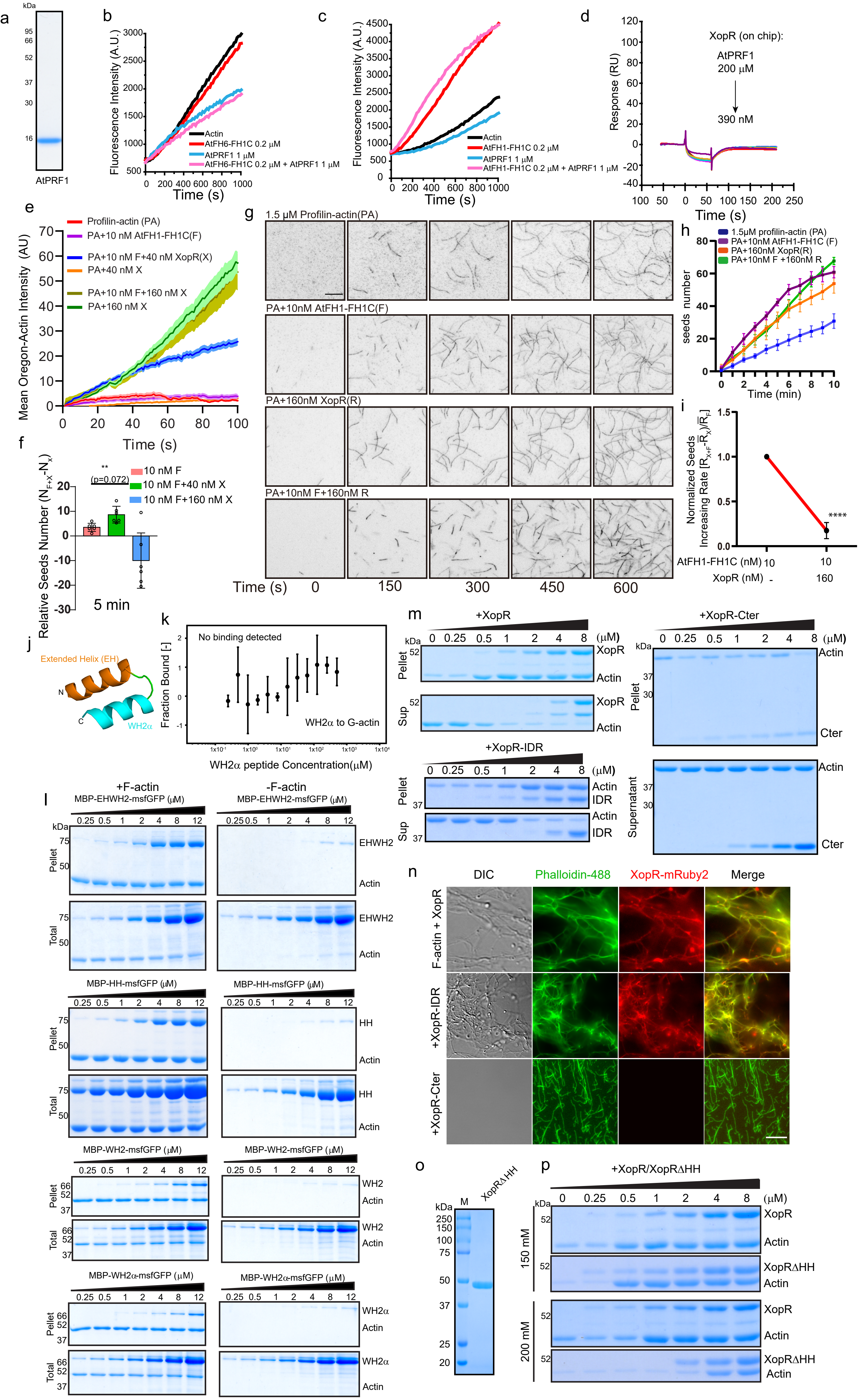
a
kDa
95
66
52
37
30
16
AtPRF1
b
Fluorescence Intensity (A.U.)
Time (s)
Actin
AtFH6-FH1C 0.2 μM
AtPRF1 1 μM
AtFH6-FH1C 0.2 μM + AtPRF1 1 μM
c
Fluorescence Intensity (A.U.)
Time (s)
Actin
AtFH1-FH1C 0.2 μM
AtPRF1 1 μM
AtFH1-FH1C 0.2 μM + AtPRF1 1 μM
d
Response (RU)
Time (s)
XopR (on chip):
AtPRF1
200 μM
390 nM
e
Mean Oregon-Actin Intensity (AU)
Time (s)
Profilin-actin (PA)
PA+10 nM AtFH1-FH1C(F)
PA+10 nM F+40 nM XopR(X)
PA+40 nM X
PA+10 nM F+160 nM X
PA+160 nM X
f
Relative Seeds Number ($N_{F+X}-N_X$)
**
(p=0.072)
10 nM F
10 nM F+40 nM X
10 nM F+160 nM X
5 min
g
1.5 μM Profilin-actin(PA)
PA+10nM AtFH1-FH1C(F)
PA+160nM XopR(R)
PA+10nM F+160nM R
Time (s)
0
150
300
450
600
h
seeds number
Time (min)
1.5μM profilin-actin (PA)
PA+10nM AtFH1-FH1C (F)
PA+160nM XopR(R)
PA+10nM F +160nM R
i
Normalized Seeds Increasing Rate $[R_{X+F}-\bar{R}_X)/\bar{R}_F]$
****
AtFH1-FH1C (nM) 10
10
XopR (nM) -
160
j
Extended Helix (EH)
N
C
WH2α
k
Fraction Bound [-]
No binding detected
WH2α to G-actin
WH2α peptide Concentration(μM)
l
+F-actin
-F-actin
MBP-EHWH2-msfGFP (μM)
0.25 0.5 1 2 4 8 12
kDa
Pellet
Total
EHWH2
Actin
MBP-HH-msfGFP (μM)
HH
MBP-WH2-msfGFP (μM)
WH2
MBP-WH2α-msfGFP (μM)
WH2α
m
+XopR
0 0.25 0.5 1 2 4 8 (μM)
Pellet
Sup
XopR
Actin
+XopR-IDR
IDR
+XopR-Cter
Supernatant
Cter
n
DIC
Phalloidin-488
XopR-mRuby2
Merge
F-actin + XopR
+XopR-IDR
+XopR-Cter
o
kDa
M
XopRΔHH
p
+XopR/XopRΔHH
0 0.25 0.5 1 2 4 8 (μM)
150 mM
200 mM
XopR
XopRΔHH
Actin

Supplementary Fig. 6 XopR regulates formin-mediated actin polymerization, F-actin binding, and bundling.

a, Coomassie-blue staining of recombinant AtPRF1 by SDS-PAGE. **b,c**, Polymerization kinetics of pyrene-actin assay for AtFH6-FH1C **b** and AtFH1-FH1C **c**, in the absence or presence of AtPRF1, at the indicated concentration. A.U. = arbitrary units. **d**, Representative SPR sensorgrams of XopR and AtPRF1. XopR was coupled to the sensor chip and injected by a serial concentration of AtPRF1 ranging from 390 nM to 200 μM. No detectable change in response unit (RU) was observed. **e**, Quantification of Oregon-actin mean intensity of 22x 22 μm^2 TIRF images from Fig 4a (n=6, data are presented as mean values with error bands which represent SD). **f**, Comparison of actin seeds number at 5min after imaging using 22x 22 μm^2 TIRF images from Fig 4a (n=6). **g**. Representative time-lapse TIRFM images of actin polymerization. 1.5 μM G-actin (10% Oregon-actin) was incubated with 6 μM AtPRF1 and the indicated concentrations of AtFH1-FH1C and XopR. **h**. Quantification of actin seeds number in the 22 x 22 μm^2 ROIs in **g** (n=6, Error bar, SD). **i**. Normalized rate changes of actin nucleation were calculated from **h** (Error bar, SD). **j**, Structure prediction of the EHWH2 region using the PEP-FOLD3 algorithm. **k**, Microscale thermophoresis binding assay of LatB-G-actin titrated with XopR WH2α peptide from experiments of three biological replicates. **l**, High-speed co-sedimentation assay of MBP-CCWH2-msfGFP, MBP-CC-msfGFP, MBP-WH2-msfGFP, and MBP-WH2α-msfGFP with 2 μM F-actin. **m**, Low-speed co-sedimentation assay of 2 μM F-actin with indicated concentrations of XopR, XopR-IDR, or XopR-Cter. **n**, F-actin forms bundles by incubating 0.2 μM F-actin with XopR (10 μM, 10% XopR-mRuby2), XopR-IDR (10 μM, 10% XopR-IDR-mRuby2), but not XopR-Cter (10 μM, 10% XopR-Cter-mRuby2). F-actin was stained by acti-488 phalloidin dye. **o**, Coomassie-blue staining of recombinant XopRΔHH by SDS-PAGE. **p**, Low-speed cosedimentation assay of purified XopR or XopRΔHH with 2 μM F-actin. A two-tailed Student's t-test was performed. *$p<0.05$, **$p<0.01$, ***$p<0.001$, ****$p<0.0001$. Scale bar = 5 μm.

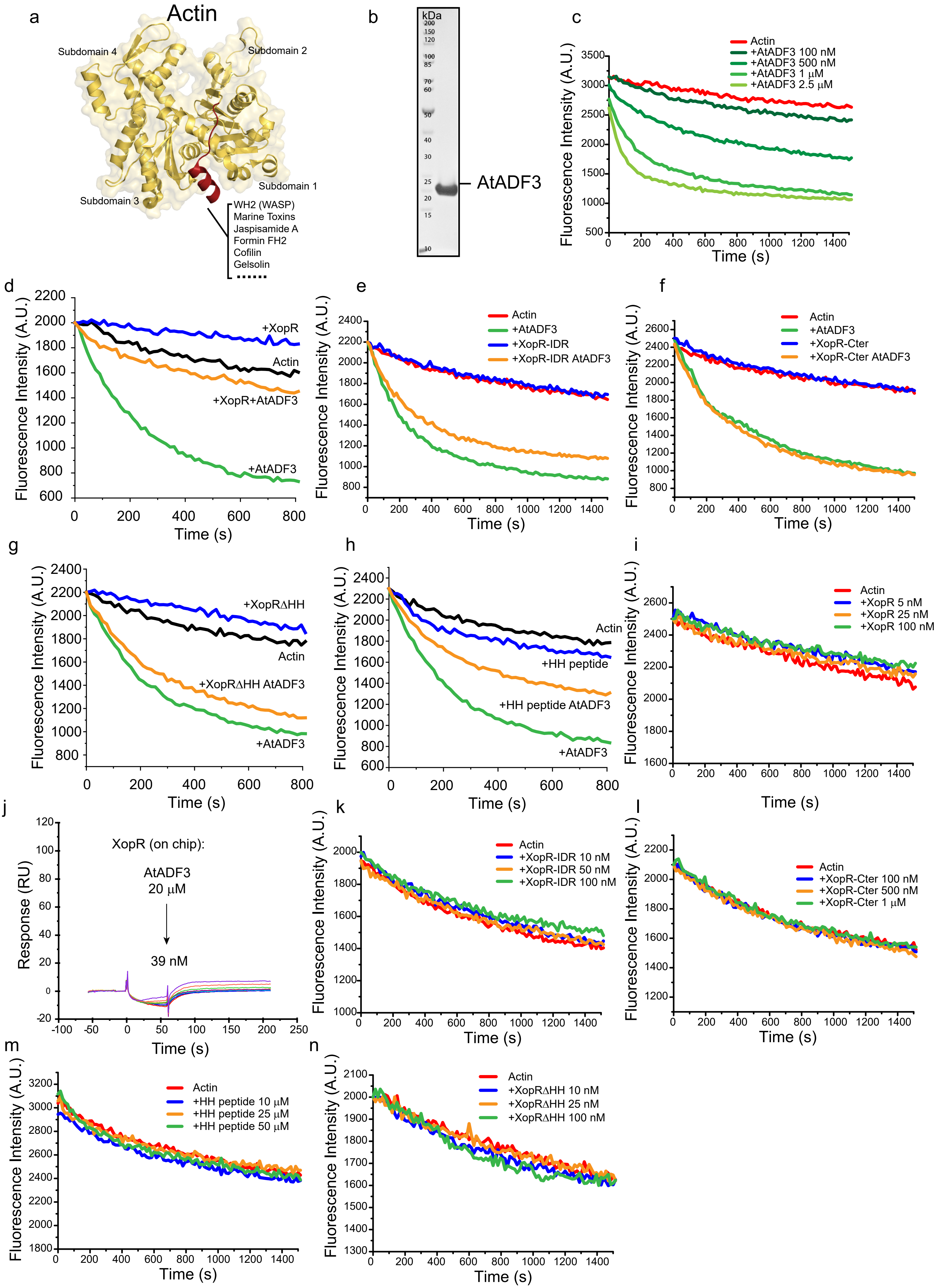

a
Actin
Subdomain 4
Subdomain 2
Subdomain 1
Subdomain 3
WH2 (WASP)
Marine Toxins
Jaspisamide A
Formin FH2
Cofilin
Gelsolin
b
kDa
200
150
120
100
85
70
60
50
40
30
25
20
15
10
AtADF3
c
Actin
+AtADF3 100 nM
+AtADF3 500 nM
+AtADF3 1 μM
+AtADF3 2.5 μM
Fluorescence Intensity (A.U.)
Time (s)
d
+XopR
Actin
+XopR+AtADF3
+AtADF3
Fluorescence Intensity (A.U.)
Time (s)
e
Actin
+AtADF3
+XopR-IDR
+XopR-IDR AtADF3
Fluorescence Intensity (A.U.)
Time (s)
f
Actin
+AtADF3
+XopR-Cter
+XopR-Cter AtADF3
Fluorescence Intensity (A.U.)
Time (s)
g
+XopRΔHH
Actin
+XopRΔHH AtADF3
+AtADF3
Fluorescence Intensity (A.U.)
Time (s)
h
Actin
+HH peptide
+HH peptide AtADF3
+AtADF3
Fluorescence Intensity (A.U.)
Time (s)
i
Actin
+XopR 5 nM
+XopR 25 nM
+XopR 100 nM
Fluorescence Intensity (A.U.)
Time (s)
j
XopR (on chip):
AtADF3
20 μM
39 nM
Response (RU)
Time (s)
k
Actin
+XopR-IDR 10 nM
+XopR-IDR 50 nM
+XopR-IDR 100 nM
Fluorescence Intensity (A.U.)
Time (s)
l
Actin
+XopR-Cter 100 nM
+XopR-Cter 500 nM
+XopR-Cter 1 μM
Fluorescence Intensity (A.U.)
Time (s)
m
Actin
+HH peptide 10 μM
+HH peptide 25 μM
+HH peptide 50 μM
Fluorescence Intensity (A.U.)
Time (s)
n
Actin
+XopRΔHH 10 nM
+XopRΔHH 25 nM
+XopRΔHH 100 nM
Fluorescence Intensity (A.U.)
Time (s)

Supplementary Fig. 7 XopR inhibits ADF-mediated F-actin depolymerization.

a, Structure of the WASP WH2-bound G-actin (PDB code: 2A3Z). WH2 domain interacts with G-actin between subdomains 1 and 3. Other partners binding to the same position are listed. **b**, Coomassie-blue staining of recombinant AtADF3 by SDS-PAGE. **c**, AtADF3 mediated actin depolymerization (30% pyrene labeled) using the indicated concentrations of AtADF3, in the presence or absence of XopR truncating variants. **d-I**, Pyrene actin depolymerization assay (30% pyrene labeled) with or without 1 μM AtADF3, in the presence of 100 nM recombinant XopR truncating variants, or 25 μM HH peptides. **j**, Representative SPR sensorgrams of XopR (on-chip) and AtADF3. A serial concentration of AtADF3 ranging from 39 nM to 20 μM was injected and showed no detectable interaction with XopR. **k-n**, Pyrene actin depolymerization assay (30% pyrene labeled) with the XopR variants at the indicated concentration.

Primer	Sequence
SHp212-XopR-FL-nostop-F	GCAGAGAACCTCTACTTC
SHp213-XopR-FL-nostop-R	GTAGTAGCCGTTGTCGATTG
SHp214-XopR-Cter-F	GATAGTCAGCTCGAGGCA
SHp215-XopR-Cter-R	CATAGTATATCTCCTTCTTAAGGTTAAAC
SHp216-XopR-FL-Ruby-vector-F	GCAGAGAACCTCTACTTCCAATCG
SHp217-XopR-FL-Ruby-vector-R	GTAGTAGCCGTTGTCGATTG
SHp218-XopR-FL-Ruby-vector-F2	GCAGAGAACCTCTACTTCCAAT
SHp219-XopR-FL-Ruby-vector-R2	GTAGTAGCCGTTGTCGATTGC
SHp220-XopR-FL-Ruby-insert-F	CAATCGACAACGGCTACTACATGGTGTCTAAGGGCGAAG
SHp221-XopR-FL-Ruby-insert-R	TGGAAGTAGAGGTTCTCTGCCTTGTACAGCTCGTCCATC
SHp222-XopR-FL-Ruby-insert-F2	CAATCGACAACGGCTACTACATGGTGTCTAAGGGCGAAGA
SHp223-XopR-FL-Ruby-insert-R2	TGGAAGTAGAGGTTCTCTGCCTTGTACAGCTCGTCCATCCC
SHp224-HHDeletion-F	GCAACTCCACAGGAGCAACACCTGC
SHp225-HHDeletion-R	GGGCTGCCGTCCGGGCTG
SHp226-AtFH1_FH1_VECTOR_R	AGAAGAACCGTGGTGATGGTGATG
SHp227-AtFH1_FH1_VECTOR_F	GGTAGTGGGAGCAACGGC

SHp228-AtFH1_FH1_INSERT_R	CTGCCGTTGCTCCCACTACCAGAAACTAATGAGATTGAGTTATGTTC
SHp229-AtFH1_FH1_INSERT_F	ACCATCACCACGGTTCTTCTCGGCAAGGTTTGCAATCTC
SHp230-AtFH6_FH1_VECTOR_R	AGAAGAACCGTGGTGATGGTGATG
SHp231-AtFH6_FH1_VECTOR_F	GGTAGTGGGAGCAACGGC
SHp232-AtFH6_FH1_INSERT_R	CTGCCGTTGCTCCCACTACCCGTAGAAGAGTTGCTGCTGTGTTCAC
SHp233-AtFH6_FH1_INSERT_F	ACCATCACCACGGTTCTTCTGCTCCGACGACGGCAGCA
SHp234-AtFH6_FH1_INSERT_F(2)	TTCTCACCATCACCACGGTTCTTCTGCTCCGACGACGGCAGCA
SHp235-WH2-F	GGCAACTCCACAGGAGCAACACCTGCTCAAGACACGGGCGGCCATTGGTAGTGGGAGCAAC
SHp236-WH2-R	TCGCGGTCCTCCATGAACAGCTGCTTTTGAATATCGCTGCACTGATTGGATTGGAAGTACAGG
SHp237-WH2a-F	ATTGGTAGTGGGAGCAAC
SHp238-WH2a-R	CTCGCGGTCCTCCATGAA
SHp239-HH-F	GTGCAGCGATATTCAAAAGCAGCTGTTCATGGAGGACCGCGAGATTGGTAGTGGGAGCAAC
SHp240-HH-R	TGCCTGGTGATCTCTTCCAGTTGCAGATCCAGGCGGCGCAACGAATTGGATTGGAAGTACAGG
SHp241-EHWH2-F	CTGCTCAAGACACGGGCGGCCATTGGTAGTGGGAGCAAC
SHp242-EHWH2-R	GTGTTGCTCCTGTGGAGTTGCCTCGCGGTCCTCCATGAA
SHp245-GFP11-F1	CGCTCTAGAACTAGTGGATCCGCAACATCCTGTGTTACGTGCG
SHp246-GFP11-R1	CGTAGTAGCCGTTGTCGATTGCC

SHp247-GFP11-GF	AATCGACAACGGCTACTACGGCGACGGTGGCTCGGGC
SHp248-GFP11-GR	CGGATCTTCAAGTAATCCCGGCGGCGTT
SHp249-GFP11-F2	CGGGATTACTTGAAGATCCGGACAGCGCA
SHp250-GFP11-R2	GATAAGCTTGATATCGAATTCGTTGATGGTGGATTGCATCACG
SHp251-HrcCD-UPF	CTATGACATGATTACGAATTCCATGCTGATGCTGCTCGGC
SHp252-HrcCD-UPR	TTGCTGCGTTCCCTCTGCGAGG
SHp253-HrcCD-DOWNF	TCGCAGAGGGAACGCAGCAACGCGTCGGGCTGCA
SHp254-HrcCD-DOWNR	CAGGTCGACTCTAGAGGATCCTTGCCTCTTCTACTTTGCGCTC
SHp255-XopRD-F	GGATTGGGTTGGCGAATTGT
SHp256-XopRD-R	AACGTCAGTGCAGCCGATTC
SHp254-XopRD-Fgene	ATGTCTTCCATCGGTGCCAG
SHp254-XopRD-Rgene	TCTCTCAGGCTCCCAAGTCA

Supplementary Table 1 List of the primers used in this study

Name	Information	References
pYM207	*pGEX4T1-GST-AtFH1-FH2-Cter-6xHIS*	Laurent Blanchoin Lab[1]
pYM1026	*Macrolab-6xHis-AtFH1-FH1-FH2-COOH-msfGFP*	This study
pYM1027	*Macrolab-6xHis-AtFH6-FH1-FH2-COOH*	This study
pYM1028	*Macrolab-6xHis-AtFH6-FH1-FH2-COOH-msfGFP*	This study
pYM1029	*pNIC-XopR-FL-6xHis-Flag*	This study
pYM1030	*pNIC-XopR-IDR-6xHis-Flag*	This study
pYM1031	*pNIC-XopR-Cter-6xHis-Flag*	This study
pYM1032	*pNIC-XopR-FL-mRuby2-6xHis-Flag*	This study
pYM1033	*pNIC-XopR-IDR-mRuby2-6xHis-Flag*	This study
pYM1034	*pNIC-XopR-Cter-mRuby2-6xHis-Flag*	This study
pYM1035	*pNIC-XopRΔHH-6xHis-Flag*	This study
pYM1036	*pNIC-AtADF3-6xHis-Flag*	This study
pYM1008	*pSY5::8xHis-HsPRF1 AmpR*	Miao Yansong Lab[2]
pYM1037	*Macrolabv-6xHis-MBP-EHWH2-msfGFP*	This study
pYM1038	*Macrolabv-6xHis-MBP-WH2-msfGFP*	This study
pYM1039	*Macrolabv-6xHis-MBP-HH-msfGFP*	This study
pYM1040	*Macrolabv-6xHis-MBP-WH2α-msfGFP*	This study
pYM1041	*pET-6xHis-superchargedGFP(-30)*	Addgene (Plasmid #62936)
pYM1042	*pK18mobsacB-HrcCΔ*	This study
pYM1043	*puj10-GFP11*	This study
pYM1044	*pER10-XopR-mRuby2*	This study

Supplementary Table 2 List of the plasmids used in this study

Supplementary Movie 1 XopR undergoes coalescence

Time-lapse DIC images over an ~53 min movie of 10 μM XopR phase separation droplet dynamics in 20 mM HEPES 50 mM NaCl pH 8 buffer. Scale bar = 10 μm.

Supplementary Movie 2 Coalescence of XopR-AtFH1-FH1C complex coaservates on the SLB

Time lapse confocal images over 140 s movie of 5 μM AtFH1-FH1C (10% Alexa647-AtFH1-FH1C) with 5 μM XopR (10% Alexa488-XopR) on SLB. Scale bar = 5 μm.

Supplementary Movie 3 Actin polymerization in the presence of formin and XopR

Time-lapse TIRF images over 600 s movie of 0.5 μM G-actin (10% Oregon-actin) polymerization in the presence of 100 nM AtFH1-FH1C and 400 nM XopR. Scale bar = 5 μm

Supplementary Reference section

1. Michelot, A. *et al.* The formin homology 1 domain modulates the actin nucleation and bundling activity of Arabidopsis FORMIN1. *Plant Cell* 17, 2296-2313 (2005).
2. Sun, H. *et al.* Profilin Negatively Regulates Formin-Mediated Actin Assembly to Modulate PAMP-Triggered Plant Immunity. *Curr Biol* 28, 1882-1895 e1887 (2018).
