# Supplementary material for: Xanthomonas effector XopR hijacks host actin cytoskeleton via complex coacervation
Source: Nat Commun. 2021 Jul 1;12:4064. doi: 10.1038/s41467-021-24375-3 (PMC8249405; doi:10.1038/s41467-021-24375-3)
Supplement: Supplementary file 3 — Description of Additional Supplementary Files [file 41467_2021_24375_MOESM3_ESM.pdf]

Description of Additional Supplementary Files

File Name: Supplementary Movie 1
Description: Time-lapse DIC images over an ~53 min movie of 10 µM XopR phase separation droplet dynamics in 20 mM HEPES 50 mM NaCl pH 8 buffer. Scale bar = 10 µm.

File Name: Supplementary Movie 2
Description: Time lapse confocal images over 140 s movie of 5 µM AtFH1-FH1C (10% Alexa647-AtFH1-FH1C) with 5 µM XopR (10% Alexa488-XopR) on SLB. Scale bar = 5 µm.

File Name: Supplementary Movie 3
Description: Time-lapse TIRF images over 600 s movie of 0.5 µM G-actin (10% Oregon-actin) polymerization in the presence of 100 nM AtFH1-FH1C and 400 nM XopR. Scale bar = 5 µm
