# Supplementary material for: Xanthomonas effector XopR hijacks host actin cytoskeleton via complex coacervation
Source: Nat Commun. 2021 Jul 1;12:4064. doi: 10.1038/s41467-021-24375-3 (PMC8249405; doi:10.1038/s41467-021-24375-3)
Supplement: Supplementary file 7 — Reporting Summary [file 41467_2021_24375_MOESM7_ESM.pdf]

nature research

Corresponding author(s): Yansong Miao

Last updated by author(s): May 10, 2021

Reporting Summary

Nature Research wishes to improve the reproducibility of the work that we publish. This form provides structure for consistency and transparency in reporting. For further information on Nature Research policies, see our Editorial Policies and the Editorial Policy Checklist.

Statistics

For all statistical analyses, confirm that the following items are present in the figure legend, table legend, main text, or Methods section.

n/a	Confirmed	
☐	☒	The exact sample size (*n*) for each experimental group/condition, given as a discrete number and unit of measurement
☐	☒	A statement on whether measurements were taken from distinct samples or whether the same sample was measured repeatedly
☐	☒	The statistical test(s) used AND whether they are one- or two-sided<br>*Only common tests should be described solely by name; describe more complex techniques in the Methods section.*
☐	☒	A description of all covariates tested
☐	☒	A description of any assumptions or corrections, such as tests of normality and adjustment for multiple comparisons
☐	☒	A full description of the statistical parameters including central tendency (e.g. means) or other basic estimates (e.g. regression coefficient) AND variation (e.g. standard deviation) or associated estimates of uncertainty (e.g. confidence intervals)
☐	☒	For null hypothesis testing, the test statistic (e.g. *F*, *t*, *r*) with confidence intervals, effect sizes, degrees of freedom and *P* value noted<br>*Give P values as exact values whenever suitable.*
☒	☐	For Bayesian analysis, information on the choice of priors and Markov chain Monte Carlo settings
☒	☐	For hierarchical and complex designs, identification of the appropriate level for tests and full reporting of outcomes
☒	☐	Estimates of effect sizes (e.g. Cohen's *d*, Pearson's *r*), indicating how they were calculated

Our web collection on statistics for biologists contains articles on many of the points above.

Software and code

Policy information about availability of computer code

Data collection	N/A
Data analysis	IUPRED2A algorithm (https://iupred2a.elte.hu/); Clustal Omega (https://www.ebi.ac.uk/Tools/msa/clustalo/); FiJi (https://imagej.net/Fiji); GraphPad Prism 8 (https://www.graphpad.com/);Biacore T200 Evaluation software (version 3.0) ;Conserved Domain Database (CDD) CD-search analysis (https://www.ncbi.nlm.nih.gov/Structure/cdd/cdd.shtml);AMPHIPASEEK (https://npsa-prabi.ibcp.fr/cgi-bin/npsa_automat.pl?page=/NPSA/npsa_amphipaseek.html);COILS algorithm (https://embnet.vital-it.ch/software/COILS_form.html);the CIDER online server (http://pappulab.wustl.edu/CIDER/analysis/); PEP-FOLD3 algorithm (https://bioserv.rpbs.univ-paris-diderot.fr/services/PEP-FOLD3/). MST analysis by MO.Affinity Analysis software V2.1.5

For manuscripts utilizing custom algorithms or software that are central to the research but not yet described in published literature, software must be made available to editors and reviewers. We strongly encourage code deposition in a community repository (e.g. GitHub). See the Nature Research guidelines for submitting code & software for further information.

Data

Policy information about availability of data

All manuscripts must include a data availability statement. This statement should provide the following information, where applicable:

- Accession codes, unique identifiers, or web links for publicly available datasets
- A list of figures that have associated raw data
- A description of any restrictions on data availability

Source data for Fig. 1b,d,e,g; Fig. 2f,i; Fig. 3b,c,d,e,g,i; Fig. 4b,c,e; Fig 5c,f,g; Fig 6b,d; Supplementary Fig. 1c,d,f,i; Supplementary Fig. 2j, m,i; Supplementary Fig. 4b,f,h,i,l,m; Supplementary Fig. 5c,d,e,i,j; Supplementary Fig 6 e,f,h,i are provided with this paper. Other data that support the findings of this study are available from the corresponding authors on reasonable request.

April 2020

Field-specific reporting

Please select the one below that is the best fit for your research. If you are not sure, read the appropriate sections before making your selection.

- [x] Life sciences
- [ ] Behavioural & social sciences
- [ ] Ecological, evolutionary & environmental sciences

For a reference copy of the document with all sections, see nature.com/documents/nr-reporting-summary-flat.pdf

Life sciences study design

All studies must disclose on these points even when the disclosure is negative.

Sample size	No sample size calculation were performed. All the sample size was determined based on experiments, literature, and enough number for sufficient statistical analysis.
Data exclusions	No data were excluded.
Replication	At least three biological replicates were performed and all attempts replication were successful
Randomization	All samples were randomly relocated into different groups
Blinding	Single blind analysis was applied to the imaging processing and analyzing

Reporting for specific materials, systems and methods

We require information from authors about some types of materials, experimental systems and methods used in many studies. Here, indicate whether each material, system or method listed is relevant to your study. If you are not sure if a list item applies to your research, read the appropriate section before selecting a response.

Materials & experimental systems

n/a	Involved in the study	
☐	☒	Antibodies
☒	☐	Eukaryotic cell lines
☒	☐	Palaeontology and archaeology
☒	☐	Animals and other organisms
☒	☐	Human research participants
☒	☐	Clinical data
☒	☐	Dual use research of concern

Methods

n/a	Involved in the study	
☒	☐	ChIP-seq
☒	☐	Flow cytometry
☒	☐	MRI-based neuroimaging

Antibodies

Antibodies used

Mouse monoclonal Anti-FLAG M2;Sigma-Aldrich;Cat # F1804 /IRDye® 800CW Goat anti-Mouse IgG Secondary Antibody

Validation

Mouse monoclonal Anti-FLAG M2
General description: The ANTI-FLAG M2 mouse, affinity purified monoclonal antibody binds to fusion proteins containing a FLAG peptide sequence.The antibody recognizes the FLAG peptide sequence at the N-terminus, Met-N-terminus,C-terminus, and internal sites of the fusion protein.
Specificity: Binding site: N-Asp-Tyr-Lys-Asp-Asp-Asp-Asp-Lys-C Immunogen: LAG; peptide sequence DYKDDDDK Application: For highly sensitive and specific detection of FLAG fusion proteins by immunoblotting[3], immunoprecipitation (IP) [1][2], immunohistochemisty, immunofluorescence [4]and immunocyotchemistry.
Optimized for single banded detection of FLAG fusion proteins in mammalian, plant, and bacterial expression systems. Western Blotting and EIA Browse additional application references in our FLAG® Literature portal(https://www.sigmaaldrich.com/life-science/proteomics/ recombinant -protein-expression/purification-detection/flag-1 iterature.html).
Legal Information: ANTI-FLAG is a registered trademark of Sigma-Aldrich Co. LLC FLAG is a registered trademark of Sigma-Aldrich Co. LLC
IRDye® 800CW Goat anti-Mouse IgG Secondary Antibody
Immunogen: Mouse IgG paraproteins

Purity and Specificity:
Isolation of specific antibodies was accomplished by affinity chromatography using pooled mouse IgG covalently linked to agarose. Based on ELISA and flow cytometry, this antibody reacts with the heavy and light chains of mouse IgG1, IgG2a, IgG2b, and IgG3, and with the light chains of mouse IgM and IgA. This antibody was tested by dot blot and and/or solid-phase adsorbed for minimal cross-reactivity with human, rabbit, goat, rat, and horse serum proteins, but may cross-react with immunoglobulins from other species. The conjugate has been specifically tested and qualified for Western blot applications.

Applications:
Western Blot
In-Cell Western™ Assay
On-Cell Western Assay
Protein Array
Immunohistochemistry
Small Animal Imaging
Microscopy
2D Gel Detection
Tissue Section Imaging
Virus Titration Assay
Formulation
IRDye 800CW secondary antibodies are supplied as purified immunoglobulin conjugates, lyophilized in phosphate-buffered saline, pH 7.4. Protect from light. Store at 4 °C prior to reconstitution.

Each vial contains 10 mg/mL BSA (free of IgG and protease) as a stabilizer and 0.01% sodium azide as a preservative, after reconstitution. Concentration is 1.0 mg/mL when reconstituted as directed. Refer to the pack insert for details on reconstitution.
